# The thermal stability and degradation mechanism of Cu/Mo nanomultilayers

**DOI:** 10.1080/14686996.2024.2357536

**Published:** 2024-05-22

**Authors:** Jeyun Yeom, Giacomo Lorenzin, Lea Ghisalberti, Claudia Cancellieri, Jolanta Janczak-Rusch

**Affiliations:** Empa, Swiss Federal Laboratories for Materials Science and Technology, Laboratory for Joining Technologies and Corrosion, Dübendorf, Switzerland

**Keywords:** Cu/Mo nanomultilayers, annealing, X-ray diffraction, fiber texture, magnetron sputtering

## Abstract

The microstructural evolution of Cu/Mo nanomultilayers upon annealing was investigated by X-ray diffraction and transmission electron microscopy. The isothermal annealing process in the temperature ranges of 300–850°C was conducted to understand the thermal behavior of the sample and follow the transformation into a nanocomposite. Annealing at 600°C led to the initiation of grain grooving in the investigated nanomultilayer, and it degraded into a spheroidized nanocomposite structure at 800°C. The sample kept the as-deposited Cu {111}//Mo{110} fiber texture up to 850°C. The residual stress was investigated to explain microstructure changes. The activation energy of degradation kinetics of Cu/Mo nanomultilayers was determined to understand the rate-determining mechanism for the degradation of nanolaminate structures.

## IMPACT STATEMENT

This study investigates the microstructural evolution of Cu/Mo nanomultilayers during vacuum annealing up to 85°C and provides important insights into their thermal stability and degradation mechanisms for development and application.

## GRAPHICAL ABSTRACT

**Figure:**
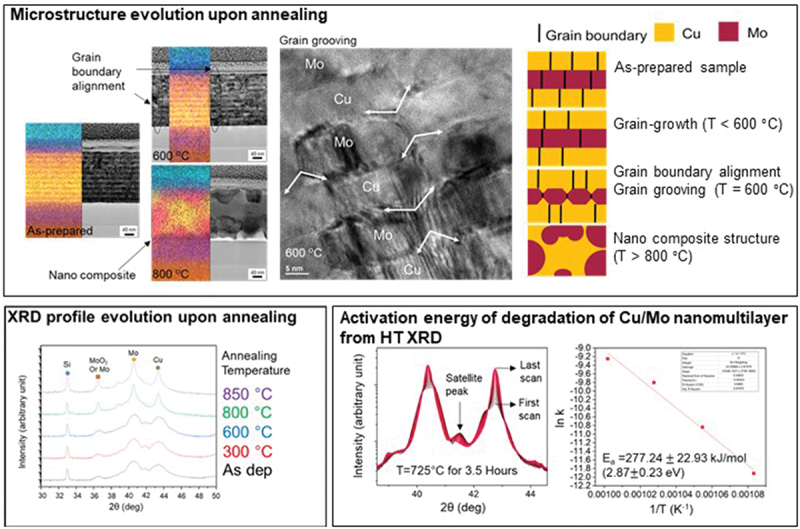

## 1. Introduction

Nanomultilayers (NMLs) also called nanolaminates or nanolamellars, are systems characterized by the repetitions of numerous layers where the thickness of each layer varies between 1 and 100 nm. The presence of a large number of interfaces, together with the possibility to combine complementary materials, can lead to a wide range of novel and outstanding properties, which differ to a great extent from those observed in monolithic films [1–4].

NMLs have been successfully produced using various methods, including accumulative roll bonding [5,6], chemical vapor deposition [7,8], electrodeposition [9,10], and physical vapor deposition (PVD) [11,12]. Among these techniques, magnetron sputtering, a form of PVD, offers distinct advantages in the synthesis of NMLs. Indeed, it enables the fabrication of a wider range of material systems as nanocrystalline configurations while providing precise control over crucial characteristics such as the number of interfaces, grain structure, and layer thickness.

The improved performances at smaller size scales exhibited by copper thin films have garnered significant interest in both fundamental science and applied research, in particular, aimed at the improvement of the reliability of microdevices [13–16]. In order to enhance the material properties of copper, extensive research has been carried out to develop advanced copper-based binary alloys, composites and multilayer systems. Specifically, notable investigations have focused on cobalt-copper (Co-Cu) [17–19], copper-tungsten (Cu-W) [20], copper-tantalum (Cu-Ta) [21], copper-chromium (Cu-Cr) [22], and copper-molybdenum (Cu-Mo) material systems [23].

In this study, we focus on Cu/Mo nanoscale metallic multilayers produced via magnetron sputtering. This system has been selected for the following reasons:

- Cu and Mo exhibit immiscibility up to relatively high temperatures. The solid solubility between bulk Cu and Mo is extremely low, at less than 1.5 wt %, even up to 900°C. As a result, annealing at moderate temperatures below 900°C can prevent the formation of Cu-Mo alloys [24].
- Cu/Mo multilayer structure represents a promising system for thermal management thanks to the combination of excellent thermal conductivity (Cu) and low coefficient of thermal expansion (Mo).

Cu-Mo-based materials have found numerous applications for their ability to collect and dissipate heat in fields, such as high-power semiconductor devices, heat sinks, electronics, and electrical engineering [25,26]. It was previously shown that these multilayers have a very low electrical resistivity [27,28].

The numerous applications demonstrate the wide-ranging utility of Cu-Mo NMLs in areas that require excellent thermal management, and high thermal and electronic conductivity. However, the high Gibbs energy resulting from the high density of interfaces poses a risk to the microstructural stability of the multilayered structure. For this reason, understanding the microstructural changes upon annealing of such laminated materials is of paramount importance, as it directly affects their performance and reliability at elevated temperatures. In this work, the microstructure evolution of Cu/Mo NMLs upon annealing up to 850°C is investigated. Texture, morphology, and residual stresses are analyzed after different heating stages. X-ray diffraction (XRD) and *in-situ* high temperature (HT) XRD experiments were carried out to understand texture evolution, interface structure changes upon annealing, and the kinetics in the underlying mechanism of NMLs degradation.

## 2. Experimental procedure

**Figure 1.**
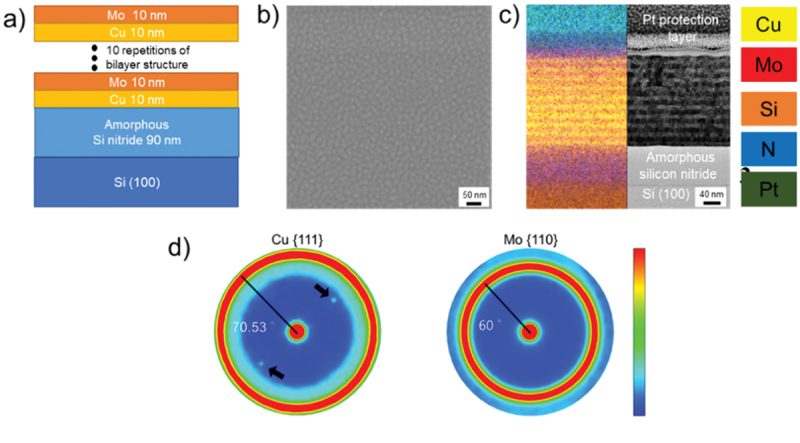
As-prepared sample: (a) illustration, (b) SEM surface image, (c) BF-STEM cross-sectional image with overlaid EDX image; the colors of the elements are indicated on the right-hand side, (d) pole figures of Cu {111} and Mo {110}.

Cu/Mo NMLs were deposited by DC magnetron sputtering on Si (001) substrates with amorphous silicon nitride on top. The thickness of the silicon nitride layer was 90 nm and it served as a barrier layer to prevent Cu diffusion into the Si substrate during the annealing process. Substrates were cleaned with acetone, ethanol, and isopropanol. Each cleaning step was 3 min long and was followed by Ar drying. RF cleaning process was carried out with 50 W power at 0.2 mbar (15 mTorr) of Ar-pressure for 2 min. DC magnetron sputtering in a high vacuum chamber (base pressure ≤ 10^−8^ mbar) was used for the deposition of Cu/Mo with gun power 80 W and Ar pressure 0.027 mbar (2 mTorr). The bilayer structure Cu_10 nm_-Mo_10 nm_ was repeated 10 times to make a total of 200 nm thick layers. (See Figure 1(a)) Isothermal annealing was carried out at temperatures 300°C, 600°C, 800°C, and 850°C for 100 min in a high vacuum environment with pressure below 10^−5^ mbar. The heating rate used to arrive at the pre-defined annealing temperature was 20 K/min.

Transmission electron microscopy (TEM) lamellas were prepared with an FEI Helios 660 Nanolab Dual-Beam Focused Ion beam/Scanning electron microscopy (FIB/SEM) system with a 30 kV Ga-ion beam. To remove the damaged layer, the cleaning process with 5 kV and 2 kV Ga-ion beams was carried out. TEM and STEM were performed using JEOL2200FS equipped with an Energy-dispersive X-ray spectroscopy (EDX) detector which was operated at 200 kV to characterize the microstructures of as-prepared samples.

A Bruker D8 Discover X-ray diffractometer was used to perform θ-2θ scans and to measure the texture of samples at room temperature and after the annealing process at 850°C. To assess the texture, pole figures were acquired around the (111) reflection of Cu and the (110) reflection of Mo. Diffraction patterns were recorded using Cu target at 40 kV and 40 mA. A modified crystallite group method introduced by Tanaka *et al*. was used for measuring residual stress in a sample with a strong fiber texture [29]. Based on the methodology of Tanaka *et al*., the residual stresses of textured Cu/Mo NMLs were analyzed under the assumption that all the components of residual stress in NMLs are equibiaxial and using the selection criteria of diffraction planes presented in the study [30].

In-situ high-temperature XRD experiments were carried out with a PANalytical X’Pert PROMPD X-ray diffractometer with a gas-tight Anton Paar XRK-900 heating chamber equipped for heating and gas feeding (5850 TR, Brooks instrument), applying an H_2_/N_2_ gas mixture of 5 vol.% H2 (99.999%, Messer) in N2 (99.999%, Messer) at a flow rate of 100 ml/min. Diffraction patterns (2θ range of 37–46º; using Cu K_α1,2_ radiation, $\lambda_{\mathrm{average}}$ = 0.15418 nm, at 40 kV and 40 mA) were recorded every ~5 min during isothermal annealing at 675, 700, and 725 º C for a total isothermal holding time of 5 h.

## 3. Results and discussion

### 3.1. Microstructure and texture of as-prepared Cu/Mo NMLs

As-prepared Cu/Mo NMLs have a Cu10nm-Mo10nm bilayer structure with 10 repetitions, as illustrated in Figure 1(a). Figure 1(b) displays the SEM surface image of the as-prepared Cu/Mo NMLs structure. The grain-like structures were formed uniformly without any cracks and voids. Figure 1(c) shows the Bright Field (BF)-STEM cross-sectional image of the sample. Here, the interface between the nanolayers can be recognized, as confirmed also by the overlayed EDX mapping image, which shows a resolved Cu/Mo NML structure.

The deposited nanolaminate structure was initially, i.e. close to the substrate interface, flat and smooth. By further increasing the thickness, the structure becomes wavy by forming polycrystalline grain-like structures, as visible in the surface image (see Figure 1(b)). Similar wavy structures have been also observed in other NML systems, such as Cu/W [31–33], Cu/Mo [34], and Cu/Nb [30].

Materials properties such as thermal conductivity [35], mechanical properties [36], and magnetic properties [37], strongly depend on the texture of materials. Understanding the texture and its changes is crucial for performing residual stress analysis using X-ray diffraction (XRD). In this regard, Figure 1d shows the pole figures of the Cu {111} and Mo {110} families of planes. The analysis reveals a preferential orientation in the layer, i.e. Cu {111}//Mo {110}, with a fiber texture. The red point at the center of both pole figures shows a high intensity, indicating an out-of-plane texture with Cu {111}//Mo {110} orientation. Additionally, the presence of in-plane random crystallographic orientations results in uniform ring structures with high intensity. The tilting angles for Cu and Mo are measured to be 70.53° and 60°, respectively, in line with the {111} and {110} textures. The two black arrows in the Cu {111} pole figure indicate a peak originating from the Si substrate.

### 3.2. Structure analysis by XRD before and after heat treatment

**Figure 2.**
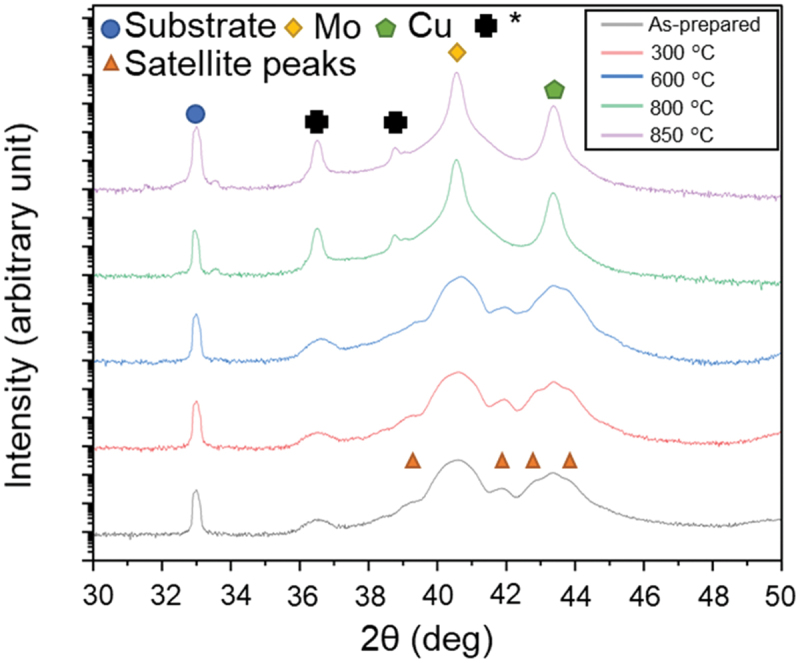
XRD θ-2θ scans of NMLs as-prepared and annealed at 300°C, 600°C, 800°C, and 850°C. The intensities were plotted on a logarithmic scale. Asterisks in the figure indicate the peaks from non-monochromatic Cu radiation.

The results of θ-2θ scans of NMLs ranging from 30° to 50° are given in Figure 2. An artificial superlattice structure from a multilayer contributes to the XRD diffraction profile producing satellite peak with producing satellite peaks [38]. The satellite peak is evident in the as-deposited NMLs and it remains till 600°C. The contribution from the superlattice structure depends on the quality of the interface structure and the disorder of the intralayer structure [39]. These satellite structures disappeared and the Full Width at Half Maximum (FWHM)s of both Cu (111) and Mo (110) peaks significantly decreased after the annealing process over 800°C. The results indicate the destruction of the periodic multilayer and the transformation into a nanocomposite (NC). The disappearance of satellite peaks in diffraction pattern and the grain grooving upon annealing has been already observed in immiscible multilayer systems at *T* > 700°C [40].

### 3.3. Microstructure and texture evolution of Cu/Mo NMLs after annealing

**Figure 3.**
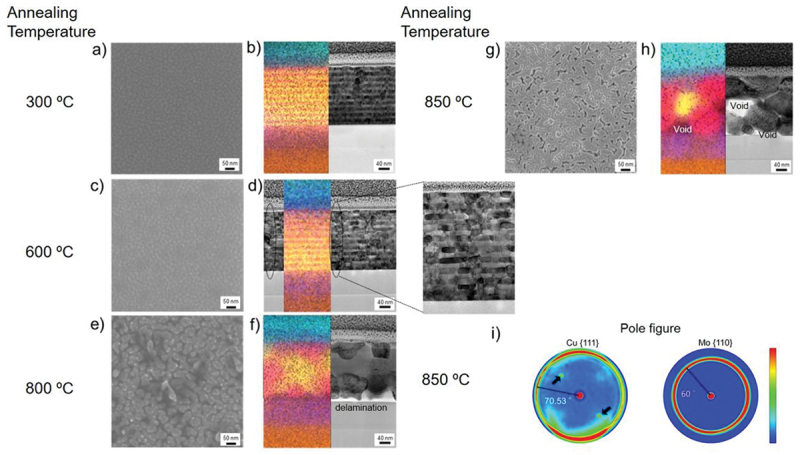
Microstructure and texture evolution of Cu/Nb NML: (a), (c), (e), (g) SEM surface images of Cu/Mo after annealing; (b), (d),(f), (g) BF-STEM cross-sectional images of with overlaid EDX images (the color of elements was kept as Figure 1); i) pole figures of Cu {111} and Mo {110} after annealing at 850°C. The dotted lines in (d) indicate aligned grain boundaries.

SEM surface images of Cu/Mo NMLs structure after annealing at 300, 600, 800, and 850°C are shown in Figures 3(a,c,e,g), respectively. The surface structure did not undergo significant modifications until annealing up to 600°C. After annealing at 800 and 850°C, the grain-like structure grew and void formation was observed in the surface structure.

BF-STEM cross-sectional images after annealing with overlapping EDX images are shown in Figs. 3b, d, f, h. Figure 3b indicates that after annealing at 300°C in-plane grain growth occurred and NMLs structure was conserved. Initiation of grain grooving was observed with the alignment of grain boundaries after annealing at 600°C and significant in-plane grain growth was verified as shown in Figure 3(d). Dotted lines in Figure 3(d) indicate aligned grain boundaries, which make a stair-like grain boundary structure (see Figure 6(a)).

Grain boundary grooving is known to initiate the degradation and pinch-off of multilayer structures with immiscible constituents leading to rapid spheroidization of the discontinuous layers [41–43]. Cu/Mo NMLs in the present work degraded to a nanocomposite structure after annealing at 800°C as shown in Figure 3(f), though there are studies reporting morphologies changes, e.g, zig-zag layered structures in Cu/Nb, multilayer structures after annealing [12,42,44,45]. The partial delamination of NC structure from amorphous silicon nitride is shown in Figure 3(f). Skeleton structure with nanocomposite and re-bonding of NC structure to substrate were observed in Figure 3(h). The void formation might result from re-bonding of NC to substrate.

The factors influencing the formation of zigzag and pinched-off microstructures in annealed NMLs were elucidated through model calculations based on the results of Cu/Mo, Cu/Ag, and Cu/Nb systems [42]. According to the model [42], the final microstructure after annealing is determined by the aspect ratio of grain dimensions and the ratio of the distance between the two nearest triple junctions to the in-plane grain size. The microstructure of Cu_5 nm_/W_5 nm_ NMLs film with 100 bilayer repetitions was examined previously and similar trends were observed: the NMLs structure completely degraded into NC structure at the same annealing temperature [40].

Figure 3(i) indicates that the crystallographic orientation of Cu/Mo was conserved exhibiting Cu {111}//Mo {110} fiber texture even though NMLs structure degraded into NC structure with voids at 850°C. This result ensures that the methodology chosen for measuring residual stress can be used for all the samples [30].

### 3.4. Residual stress analysis

**Figure 4.**
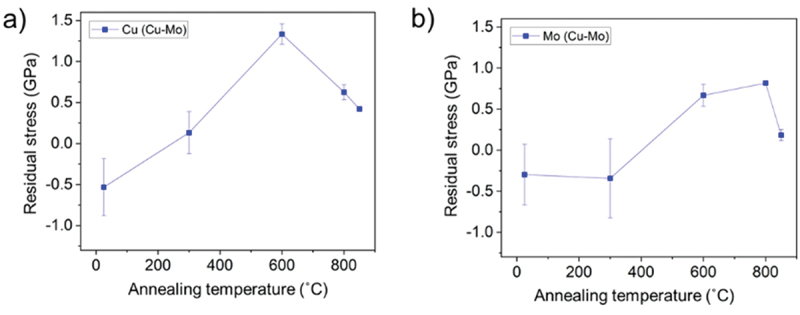
Residual stress evolution of Cu/Mo NML as-prepared and annealed at 300°C, 600°C, 800°C, and 850°C. (a) residual stress of Cu. (b) residual stress of Mo.

Figure 4 shows the residual stress evolution of Cu/Mo nanomultilayer. As-prepared NML exhibited compressive stress in both Cu and Mo layers [30]. During the annealing process, thermally induced stresses develop in the film, due to the coefficient of thermal expansion (CTE) mismatch between substrate and film material. During the initial stage of a heating or cooling segment, when the deformation is purely elastic, the stress-temperature behavior of the film follows the thermoelastic line given by

(1) $$\sigma_{\mathrm{total}}\;=\sigma_{0}+\;\frac{E_{\mathrm{film}}}{1-v_{\mathrm{film}}}\left(\alpha_{\mathrm{sub}}-\alpha_{\mathrm{film}}\right)\left(T-T_{0}\right)$$

where *σ*_0_ is the stress at the initial (room) temperature *T*_0_, *E*_film_ and *ν*_film_ are Young’s modulus and Poisson’s ratio of the film, *E*_film_/(1-v_film_) is the biaxial elastic modulus of the film, and *α*_sub_ and *α*_film_ are the CTE of substrate and film, respectively [46]. In this work, *σ*_0_ measured at room temperature was initially generated during deposition, and it was modified due to the temperature difference between the annealing temperature and room temperature. The measured stress of *σ*_0_ by XRD can be divided into coherence stress and deposition stress [47] and it was found to be compressive for the as-deposited NML. After the annealing process, residual stress measured ex-situ turned into tensile stress (see Figure 4). Both Cu and Mo exhibited tensile stress after annealing at *T* > 300°C with a maximum tensile value between 600–800°C, as measured by ex-situ XRD. Above these temperature ranges, stress relaxation occurs in both layers. To explain this turnover of stress, one has to analyze what happens during the thermal treatment. During the heating step, thermally induced compressive stress will be applied to NMLs as expected from Equation 1 (*α*_cu_ = 16.6 × 10^−6^ K^−1^, *α*_Mo_ = 5 × 10^−6^ K^−1^, *α*_Si_ = 2.6 × 10^−6^ K^−1^ [48]). At high temperatures, residual stresses in both Cu and Mo layers become compressive as the lattice expands following the thermal expansion (*α*_film_ > *α*_Si_). Then, the compressive residual stresses in both layers can be relaxed through diffusion, creep, or dislocation climb and glide [46,49]. During cooling down, tensile stress builds up in the NMLs. Tensile stress is indeed measured by XRD at room temperature, after annealing. The delamination of Cu/Mo NMLs mainly occurred near the edge of the substrate after 800°C annealing. This is not surprising since the vulnerability of film edge to delamination was reported [50,51]. The delamination can be explained by high residual stresses in the Cu nanolayers (1.3 GPa even after annealing at 600°C) [52]. The observed delamination of the Cu/Mo nanomultilayer from the Si substrate at annealing temperatures above 800°C, led to new pathways for the residual stress relief, resulting in a similar magnitude of residual stress in the annealed Cu and Mo layers as observed after annealing at 850°C.

As previously mentioned, the sample surface morphology also transforms during the thermal treatment (See Figure 3). Hillocks growth (i.e. protrusions of material on the surface) would be expected for this system as Cu hillock growth in Mo/Cu multilayer on Si substrate was already reported [48]. Moreover, several models were introduced to explain hillock growth, and a common factor in all of them was the requirement of compressive stress in the plane of a film [53]. However, there is no evidence of Cu hillock growth in the Cu/Mo NMLs samples in this work at any used annealing temperature, though a similar immiscible system of Cu/W NMLs on Si substrate exhibited important Cu hillock growth at about 500°C annealing temperature [54]. The occurrence of Cu surface outflow may be influenced by the difference in magnitudes of initial compressive residual stresses. The compressive residual stresses of Cu and Mo in Cu/Mo are considerably lower than those of Cu and W in Cu/W [54]. Considerably high compressive growth stress on the hard W (>3 GPa in magnitude) compared to less than 0.5 GPa in Mo monolayers influences the softer Cu which adapts to the W layer and releases its stress by surface outflowing upon annealing [54].

### 3.5. Grain grooving of Cu/Mo NMLs and kinetics of NC structure formation

**Figure 5.**
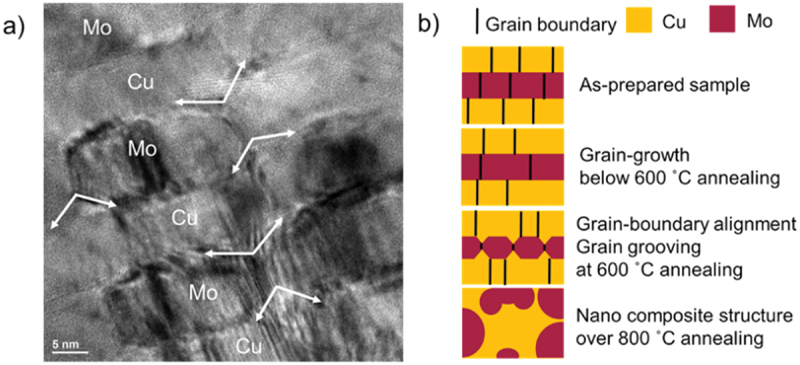
(a) HR-TEM cross-sectional image of Cu/Mo NML annealed at 600°C, (b) illustration of degradation mechanism of Cu/Mo NML.

The morphology of grain grooving in equilibrium can be expressed as follows:

(2) $$2\cos\frac{\theta }{2}=\;\frac{\gamma_{\mathrm{gb}}}{\gamma_{\mathrm{int}}}$$

where θ is the grooving angle of the grain boundary (see Figure 5(a)), $\gamma_{\mathrm{gb}}$ is grain boundary energy. The Equation (2) assumes isotropic interface energies and mechanical equilibrium [55]. If the ratio of $\frac{\gamma_{\mathrm{gb}}}{\gamma_{\mathrm{int}}}$ becomes large, the grooving angle becomes small leading to pinch-off.

Though, grain grooving occurred in both, Cu and Mo cases, deeper grain boundary grooving was observed in Mo which will lead to pinch-off as shown in Figure 5(a). This is because the free energies of the grain boundary can be scaled with the melting temperature [56], and the melting temperature of Mo is higher than that of Cu. A similar result was obtained in Cu/Ta NMLs where deeper grain boundary grooving in Ta was observed leading to pinch-off [43]. Figure 5(b) summarizes in a schematic representation the morphological change of Cu/Mo NMLs upon annealing. The grooving angles of Mo in the Cu nanolaminate matrix are presented in Table 1.

**Table 1.** The grooving angle of Mo grain boundary in the Cu nanolaminate matrix.

	$\theta_{1}$	$\theta_{2}$	$\theta_{3}$	$\theta_{4}$	$\theta_{5}$
Grooving angle	115°	111°	130°	126°	102°

Pinch-off of NMLs can be prevented by controlling in-plane grain size and layer thickness as explained by references [42,45]. The theoretical predictions need interface energy and grain boundary energy of Cu/Mo NMLs. However, the interface energy of Cu/Mo has been scarcely discussed, despite its importance in designing stable Cu/Mo NML structures. Based on Table 1, Equation (2), and the grain boundary energy of Mo [57], the interface energy of Cu/Mo is estimated approximately 0.5 ~ 2.0 $J/m^{2}$ at 600°C. Recently, by using density functional theory (DFT), the interface energy of Cu/Mo was calculated for four distinct structures, yielding in a value range from 3.09 to 4.11 $J/m^{2}$ [58]. The experimentally estimated interface energy is smaller than the calculated value. Given that a sharp rise in interface energy is observed as the temperature decreases [59], this discrepancy can be explained by the fact that the Density functional theory (DFT) calculation assumes 0 K.

**Figure 6.**
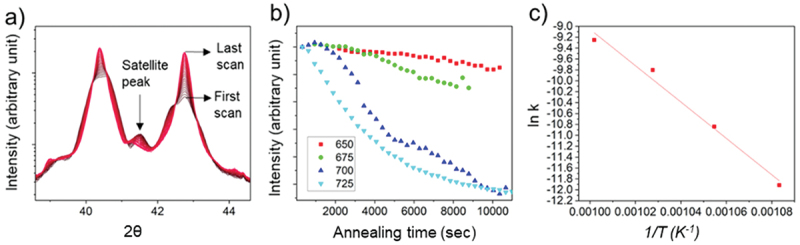
In-situ HT-XRD results. (a) θ-2θ scans as a function of isothermal holding time exemplarily shown for an annealing temperature fixed at 700°C (only scans after every 5 min are shown for clarity). The intensity is plotted on a logarithmic scale. A multi-peak fit procedure was applied to deconvolute the integrated intensity of the different satellite peaks. (b) Normalized intensity of satellite (I/I_0_) as a function of annealing time for the different holding temperatures investigated (650 ~ 725 °C). The intensity is plotted on a linear scale. (c) it shows an Arrhenius plot deduced from stage II. The activation energy was evaluated using the slope.

As the superlattice structure of NMLs contributes to additional XRD peaks, the degradation of the multilayer structure and its kinetics upon annealing can be investigated by monitoring satellite peak intensity in-situ HT XRD [40,60]. When NML transforms into an NC, satellite peaks disappear as the layer periodicity along the z direction is lost. The ending results are single diffraction peaks of individual Mo and Cu components. Figure 6(a) exemplarily shows θ-2θ scans as a function of isothermal-holding time at a fixed annealing temperature of 700°C. Each profile was obtained every 5 min, changing the color from dark red to bright red. At the end of the isothermal treatment, independently of the temperature used, higher peak intensity of Cu (111) and Mo (110) reflections was observed. The satellite peaks, present at the beginning, gradually disappeared as annealing time elapsed. Figure 6(b) shows the peak intensity change of the satellite peak indicated in Figure 6(a). The evolution of other satellite peak intensity follows a similar trend confirming the reliability of the method used. For the first ~2000 sec of the isothermal annealing, more pronounced for *T* < 725°C, an increase of satellite peak intensity was observed. This was also reported in a previous work [40], and it was attributed to a reduced roughness at the interfaces upon annealing. A lowered interface disorder increases the satellite peak intensity in a superlattice. After this stage, the overall satellite peak intensity begins to decrease linearly with time, indicating the onset of the multilayer degradation into NC. The activation energy of NMLs thermal degradation was calculated by linear regression analysis in the Arrhenius plot (See Figure 6(c)). The activation energy is found to be 277.24 ± 22.93 kJ/mol (2.87±0.23 eV). Table 2 summarizes reported activation energies for grain boundary, surface, and lattice self-diffusion of Cu, and Mo. The activation energy of degradation of NMLs matches well with grain boundary and surface diffusion of Mo. The diffusion of Cu is much faster than Mo due to a much lower melting temperature. Therefore, the rate-determining step for the degradation of Cu/Mo NMLs is the diffusion process of Mo along grain boundaries or phase boundaries. In the case of Cu/W multilayers, it was also found that the activation energy was in the range of W surface and grain boundary diffusion [40]. Both results on immiscible metallic multilayers validate the theory that mobility of the higher melting point metal, W or Mo, along the grain and interfacial boundaries, is the rate-limiting mechanism for NML degradation.

**Table 2.** Reported activation energies for grain boundary, surface, and lattice self-diffusion of Cu, and Mo.

Diffusion pathway	Cu (kJ/mol)	Ref.	Mo (kJ/mol)	Ref.
Grain-boundary	62	[61]	188–282*	
Surface	75–87	[62]	203–264	[63]
Lattice	211	[64]	375–423	[65]

*According to a reference [66], the activation energy for grain boundary diffusion of bcc transition metals can be assumed to be 1/2 to 2/3 of the activation energy for lattice diffusion.

## 4. Summary and conclusion

The microstructure evolution of Cu/Mo NMLs was investigated upon vacuum annealing (300–850 °C). Annealing at 600°C led to the initiation of grain grooving in the NML, which then degraded into a spheroidized nanocomposite structure and partially delaminated from the substrate at 800°C. By annealing at 850°C, void structures were formed, most possibly due to re-bonding to the amorphous silicon nitride substrate. The prepared sample kept Cu {111}//Mo{110} fiber texture, up to annealing at 850°C. Residual stresses of Cu/Mo nanomultilayer were analyzed to explain the degradation mechanism. No evidence of Cu hillock growth was observed. The interface energy of Cu/Mo is estimated approximately 0.5 ~ 2.0 $J/m^{2}$ at 600°C. The activation energy of degradation of Cu/Mo NMLs was determined as 277.24 ± 22.93 kJ/mol (2.87±0.23 eV), indicating that the rate-determining step for the degradation of NML is the diffusion process of Mo along grain boundaries or phase boundaries. These findings play a crucial role in enhancing our understanding of the thermal stability and degradation mechanism of Cu/Mo nanomultilayers, which pave the way to support the development of nanolayered materials with improved performance.
